# Contributions of cardiac “funny” (f) channels and sarcoplasmic reticulum Ca^2+^ in regulating beating rate of mouse and guinea pig sinoatrial node

**DOI:** 10.14814/phy2.12561

**Published:** 2015-12-10

**Authors:** Islom B. Nazarov, Christopher J. Schofield, Derek A. Terrar

**Affiliations:** ^1^ Department of PharmacologyUniversity of OxfordOxfordUK; ^2^ Department of ChemistryChemistry Research LaboratoryUniversity of OxfordOxfordUK

**Keywords:** Calcium, cardiac, HCN, I(f), pacemaker, sarcoplasmic reticulum, sino‐atrial node

## Abstract

The aim of this study was to investigate the effects on spontaneous beating rate of mouse atrial preparations following selective block of cardiac “funny” (f) channels, I(f), and/or suppression of sarcoplasmic reticulum (SR) function in the absence and presence of *β*‐adrenoceptor stimulation. ZD7288 [to block I(f)] caused a substantial reduction (222 ± 13 bpm) in beating rate from 431 ± 14 to 209 ± 14 bpm, ryanodine alone (to block SR Ca^2+^ release) reduced beating rate by 105 ± 11 bpm, with subsequent addition of ZD7288 further reducing rate by 57 ± 9 bpm. Cyclopiazonic acid (CPA) alone (to inhibit Ca^2+^ reuptake by the SR) reduced beating rate by 148 ± 13 bpm with subsequent addition of ZD7288 further reducing rate by 79 ± 12 bpm. In additional experiments measuring Ca^2+^ transients in the SA node region using Rhod‐2, effects of ivabradine and ZD7288 on rate were again substantially reduced after CPA. Effects of CPA alone on rate developed much more slowly than effects on Ca^2+^ transient amplitude. ZD7288, ivabradine, and CPA reduced the slope and maximum response of the log(concentration)–response curves for effects of isoprenaline on beating rate. Very little response to isoprenaline remained after treatment with CPA followed by ZD7288. Similar changes in isoprenaline log(concentration)–response curves were seen in guinea pig preparations. These observations are consistent with a role for Ca^2+^ released from the SR in regulating I(f) and therefore beating rate of SA node preparations; there appear to be additional contributions of SR‐derived Ca^2+^ to effects of *β*‐adrenoceptor stimulation on beating rate that are independent of I(f).

## Introduction

Despite its medical importance, the mechanisms underlying regulation of spontaneous heart rate by specialized muscle cells in the natural pacemaker of the sinoatrial node remain controversial. Our work has highlighted the importance of mechanisms dependent on Ca^2+^ release from the sarcoplasmic reticulum (SR) both in the absence and presence of *β*‐adrenoceptor stimulation (Rigg and Terrar 1996; Rigg et al. 2000; see also Vinogradova et al. 2002). Lakatta and coworkers have emphasized the importance of a “calcium clock” arising from mechanisms involving spontaneous calcium release from the SR (Vinogradova et al. 2004), suggesting that such a “calcium clock” might exert a dominant influence over a “membrane clock” (in which the timing mechanism is dependent on the sequential activation and deactivation of membrane ion channels), though recent work proposes a coupled‐clock mechanism involving integration of both calcium and membrane clocks (e.g., Lakatta et al. 2010; Yaniv et al. 2015). DiFrancesco has debated the importance of the calcium clock with Lakatta, and attributes a major role to the I(f) current activated by hyperpolarization and regulated by cAMP (Lakatta and DiFrancesco 2009; DiFrancesco 2010). Work from Noma and coworkers has led them to question the role of cytosolic Ca^2+^, including that released from the SR, emphasizing the dominance of the membrane clock but assigning a less important role to I(f), at least in the absence of *β*‐adrenoceptor stimulation (Himeno et al. 2011; although see Capel and Terrar 2015a). The diversity of Ca^2+^‐dependent mechanisms for the control of pacemaker activity has been recently reviewed (Capel and Terrar 2015b). The purpose of the work presented here was to use drugs to assess the importance of these mechanisms both in the absence of autonomic or hormonal influences and in the presence of *β*‐adrenoceptor stimulation.

The protein ion channel that carries I(f) is made up of HCN (Hyperpolarization activated Cyclic Nucleotide regulated) subunits 1–4, with HCN4 probably playing the most important role in the heart. Genetic work shows that suppression of HCN4 slows but does not abolish spontaneous activity (Mesirca et al. 2014). In the case of selective I(f) inhibitors, ZD7288 (BoSmith et al. 1993) is an established experimental tool, and more recently ivabradine (a clinically used bradycardic agent) has become available (Bois et al. 1996 & Bucchi et al. 2002). We used a combination of these drugs with the aim of selectively blocking most, if not all, of the I(f) current. We also used agents to suppress SR function, ryanodine, and cyclopiazonic acid (CPA, a selective inhibitor of Ca^2+^ uptake into the SR, Seidler et al. 1989), which have both previously been shown to reduce spontaneous beating rate in the absence of other drugs and hormones (Rigg and Terrar 1996). Ryanodine also decreases the positive chronotropic effect of isoprenaline, reducing the slope and maximum response of the log(concentration)–response curve to this agonist measured in intact guinea pig sinoatrial node preparations and in single myocytes isolated from rabbit sinoatrial node (Rigg et al. 2000; Vinogradova et al. 2002). Experiments were carried out both with and without the use of Ca^2+^ probes to measure cytosolic Ca^2+^. The spontaneously beating atrial preparation, dissected free of the AV node and the underlying ventricles, was chosen for some experiments because it was thought that such experiments approximate physiological conditions (although these preparations are removed from neuronal and hormonal influences that would otherwise occur in the whole animal). For experiments in which cytosolic Ca^2+^ was to be measured using Rhod‐2, the preparations were subjected to additional dissection to reveal the translucent region in the right atrium adjacent to the crista terminalis. This translucent region contains the site of origin of pacemaker activity (see below) and was visualized with an EMCCD camera in experiments in which the amplitude and timing of Ca^2+^ transients were measured.

## Methods

All procedures involving animals were approved by the Oxford University ethics board in accordance with the Home Office (UK) Code of Practice and Animals (Scientific Procedures) Act 1986 guidelines. Schedule 1 was performed on male CD1 mice and guinea pig by initial stunning followed by cervical dislocation. The heart was rapidly excised and placed in a chamber with a Sylgard^®^ (Dow Corning) base containing oxygenated Physiological Salt Solution (PSS) at room temperature. The ventricles were dissected away leaving intact isolated atrial preparation.

### Organ bath atrial node preparations

Following the removal of ventricles, nonabsorbable braded silk‐waxed suture (Pearsalls Ltd., Taunton, MA) was used to carefully tie a basic surgical knot around the lateral wings of the two atria. The preparation was then transferred to a 20 mL organ bath containing 10 mL oxygenated PSS, warmed by a water jacket to 37°C. The atria were mounted vertically such that the left atrium was attached to a metal hook positioned at the bottom of the organ bath and the right atrium was attached to an isometric force transducer (AD Instruments, Oxford, UK). The beating rate was calculated from the interval between contractions using Chart 5 software. The beating rate was also calculated using an extracellular electrode placed close to the SAN region to record a compound action potential signal. The beating rate was calculated from the interval between individual extracellular voltage signals associated with compound action potentials using Chart 5 software. Thirty‐minute incubation of the tissue with SR inhibitors, MDL‐12330A, ivabradine, and ZD7288 was required to reach a reduced plateau steady beating rate. The rate (beats per minute) was measured from beats sampled over a period of approximately 20 sec. The preparation was allowed to stabilize for 45 min before an experiment was carried out.

### Optical Ca^2+^ transient measurement sinoatrial node preparation

Vessels and connective tissue from the external surface of the isolated atrial preparation surrounding the superior vena cava were carefully removed without interfering with the posterior wall of the node. The preparation was then transferred to a 20‐mL chamber (with Sylgard base) containing 10 mL of oxygenated PSS. Next the atrial preparation was pinned to the Sylgard base and positioned so that the left atrium was on the right with the superior vena cava at the top. The right atrium was opened in order to expose the sinoatrial node area by making incision from the bottom of the right atrium toward the superior vena cava. This in turn exposed crista terminalis bands from the left‐hand side of the right atrium. Anatomically the sinoatrial node region differs from atrial tissue by being translucent and positioned close to the crista terminalis and away from the interatrial septum. The exposed beating atrial preparations were then kept at 34°C (to reduce the extrusion of the Ca^2+^ probe) and incubated in PSS for 30 min with 10 *μ*mol/L blebbistatin, followed by 45 min incubation with 4 *μ*mol/L Rhod‐2, AM. The solution was then replaced with PSS containing 10 *μ*mol/L blebbistatin; the preparation was allowed to stabilize for 10 min before experimentation. The location of the leading pacemaker site was observed as a stable fluorescence signal resulting from excitation of Rhod‐2, originating from right atrium site near the superior vena cava where the SAN is anatomically defined. Upon detection of the leading pacemaker site we selected region of interest corresponding to an area of 375 by 375 *μ*m from its central core to record Ca^2+^ transients. Under the conditions of our experiments the leading pacemaker site remained stable during application of drugs. Thirty‐minute incubation of the tissue with SR inhibitors and ZD7288 was required to reach a reduced plateau steady beating rate. The recording was sampled over 10 sec at a frame rate of 3000 frame/sec. The above procedures did not interfere with the spontaneous rhythm or cause any irregularities of the rhythm. In particular, little or no change in heart rate was detected by a surface electrode to record extracellular compound action potentials following application of blebbistatin and Rhod 2 (see Fig. 4C).

### Imaging system for Ca^2+^ transient measurements

For illumination, we used four green LEDs with band‐pass filters, 560/50 nm, to excite the Ca^2+^ sensitive dye (Rhod‐2 AM). Emission filter, ET585/40 nm, was used for the collection of emitted fluorescent (purchased from Cairn Research Ltd., Kent, UK). An EMCCD camera with 128 × 128 pixels (Evolve 128 Photometrics, Tucson) was used for optical recordings. The sinoatrial node preparation was exposed to light only during image acquisition. The acquired fluorescent signal was visualized and quantified using custom software kindly provided by Gil Bub (Department of Physiology, Anatomy and Genetics, University of Oxford). Ca^2+^ transients were analyzed using the pCLAMP software.

### Solutions and chemicals

Physiological salt solution (PSS) was prepared daily and contained (mmol/L) 125.0 NaCl, 25.0 NaHCO_3_, 5.4 KCl, 1.2 NaH_2_PO_4_, 1.0 MgCl_2_, 5.5 glucose, 1.8 CaCl_2_, and the solution was aerated with 95% O_2_/5% CO_2_ to maintain a pH of 7.4. ZD7288, ryanodine, MDL12330A, and ivabradine were from Tocris Inc. Rhod‐2, AM, was from Life Sciences Technologies. All other chemicals were from Sigma Co. Except for isoprenaline and ryanodine, which were dissolved in distilled water, all other chemicals were dissolved in dimethyl sulfoxide (DMSO).

### Drug exposure times

For ZD7288, ivabradine, ryanodine, and MDL12330A an exposure time of at least 30 min was allowed, since it was found that there was a slow decline of spontaneous rate to a plateau steady state within this period. Spontaneous beating was generally regular during exposure to drugs, and any periods of irregularity were avoided for measurements of spontaneous rate.

### Statistical data analysis

Results are expressed as means ± SEM, *n *= number of experiments. GraphPad prism (version 5.0) software was used to perform statistical analysis including Student's *t*‐test and two‐way analysis of variance (ANOVA), Bonferroni posttest (significance level, *P *<* *0.05).

## Results

### Effects of I(f) blockade and suppression of SR function on spontaneous beating rate in the absence of β‐adrenoceptor stimulation

**Figure 1:**
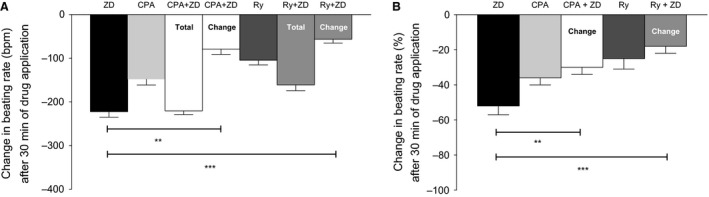
(A) The effect of I(f) inhibition on the spontaneous beating rate of mouse atrial preparations by 1 *μ*mol/L ZD7288 (*n *= 9), ryanodine receptor inhibition by 30 *μ*mol/L ryanodine (*n *= 6), and by the combined application of 30 *μ*mol/L ryanodine + 1 *μ*mol/L ZD7288 (*n *= 6, Total represents cumulative change from baseline, while Change represents a relative change after ryanodine), SERCA inhibition by 100 *μ*mol/L CPA (*n* = 7) and combined application of 100 *μ*mol/L CPA + 1 *μ*mol/L ZD7288 (*n* = 6, Total represents cumulative change from baseline, while Change represents a relative change after CPA). (B) Data presented as percentage change relative to baseline (except for 30 *μ*mol/L ryanodine + 1 *μ*mol/L ZD7288, and 100 *μ*mol/L CPA + 1 *μ*mol/L ZD7288 combinations as the % changes are calculated relative to reduced baseline by ryanodine and CPA, respectively). Data are expressed as mean ± SEM,* n* = number of experiments. GraphPad prism (version 5.0) software was used to perform statistical analysis including Student's *t*‐test (significance level, *P *< 0.05).

Initially, we used ZD7288 (1 *μ*mol/L) to investigate the effect of I(f) inhibition on spontaneously beating mouse atrial preparations (organ bath experiments in the absence of Ca^2+^ probe). The tissue was incubated with ZD7288 for 30 min, and during this time the beating rate reached a new lower steady state. Inhibition of I(f) with 1 *μ*mol/L ZD7288 caused an approximate halving of the spontaneous beating rate. The spontaneous beating rate was reduced by 52 ± 5%, a reduction of 222 ± 13 bpm from a resting beating rate of 431 ± 14 bpm to 209 ± 14 bpm (*P < *0.05, *n *=* *9, Fig. 1).

To evaluate the contribution of Ca^2+^ release from the SR on the spontaneous beating rate, we then used either ryanodine (to block Ca^2+^ release) or cyclopiazonic acid (CPA, to suppress SR function by inhibiting Ca^2+^ uptake). Thirty‐minute incubation of the tissue with SR inhibitors was required to reach a reduced plateau steady beating rate. In the case of 30 *μ*mol/L ryanodine, the spontaneous beating rate was reduced by 25 ± 6%, a reduction of 105 ± 11 bpm from a resting beating rate of 425 ± 18 bpm to 320 ± 17 bpm (*P *<* *0.05, *n *=* *6). With 100 *μ*mol/L CPA, the spontaneous beating rate was reduced by 36 ± 4%, a reduction of 148 ± 13 bpm from 413 ± 19 bpm to 265 ± 14 bpm (*P *<* *0.05, *n *=* *7).

We then investigated the effect of ZD7288 in slowing the spontaneous beating rate, but with initial suppression of Ca^2+^ release from SR (either with ryanodine or CPA). Compared with the 222 ± 13 bpm slowing reported above, the effects of ZD7288 on spontaneous beating rate after ryanodine or CPA appeared to be less than in the absence of drugs to suppress SR function: after ryanodine the change in beating rate was 57 ± 9 bpm (*P *<* *0.05 for comparison of effects of ZD7288 in the presence and absence of ryanodine, *n *=* *6 for both datasets) and after CPA the change in beating rate was 79 ± 12 bpm (*P *<* *0.05, for comparison of effects of ZD7288 in the presence and absence of CPA, *n *=* *6 for both datasets). Thirty‐minute incubation of a tissue with ZD7288 was performed after the 30‐min preincubation with SR inhibitor to reach a further reduced plateau steady‐state beating rate.

The results of these experiments provide support for the hypothesis that the effects of blockade of I(f) on spontaneous rate are less when SR function is suppressed, perhaps because this leads to changes in cytosolic Ca^2+^ that affects Ca^2+^‐sensitive adenylyl cyclases (Mattick et al. 2007). These enzymes can regulate cAMP and therefore influence I(f) (see Discussion).

**Figure 2:**
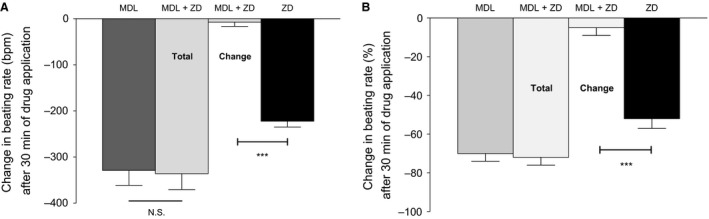
(A) The effect of AC inhibition by 10 *μ*mol/L MDL12330A (*n* = 6), 1 *μ*mol/L ZD7288 (*n *=* *9), or by the combination of 1 *μ*mol/L ZD7288 in the presence of 10 *μ*mol/L MDL12330A (*n *=* *6, Total represents cumulative change from baseline, while Change represents a relative change after MDL), on the spontaneous beating rate of mouse atrial preparations. (B) Data presented as percentage change. Data are expressed as mean ± SEM,* n *= number of experiments. GraphPad prism (version 5.0) software was used to perform statistical analysis including Student's *t*‐test (significance level, *P *< 0.05). Note that observations on the actions of ZD alone from Figure 1 are included to facilitate comparison.

Adenylyl cyclases (including those stimulated by cytosolic Ca^2+^) can be inhibited by MDL12330A (Grupp et al. 1980; Kanda and Watanabe 2001). We therefore investigated whether the slowing effect of ZD7288 on spontaneous beating rate might be influenced by pretreatment of the preparation with 10 *μ*mol/L MDL12330A (allowing 30‐min incubation for the beating rate to be reduced to a plateau steady beating rate). Exposure to MDL12330A reduced the basal beating rate from 443 ± 24 bpm to 131 ± 20 bpm (a reduction of 70 ± 4% expressed as % resting rate, *P *<* *0.05, *n *=* *6) (Fig. 2). Under these conditions, addition of ZD7288 did not produce a statistically significant further reduction relative to MDL12330A alone (131 ± 20 to 124 ± 10 bpm, a reduction of 5 ± 4%, *P *>* *0.05, *n *=* *6). It therefore appears that the effect of ZD7288 in slowing the spontaneous beating rate after MDL12330A was substantially reduced or abolished (7 ± 8 bpm, *P *<* *0.05, for comparison of effects of ZD7288 in the presence and absence of MDL12330A, *n *=* *6 for both datasets) relative to the reduction in rate observed in the absence of MDL12330A (approximately 200 bpm as reported above).

**Figure 3:**
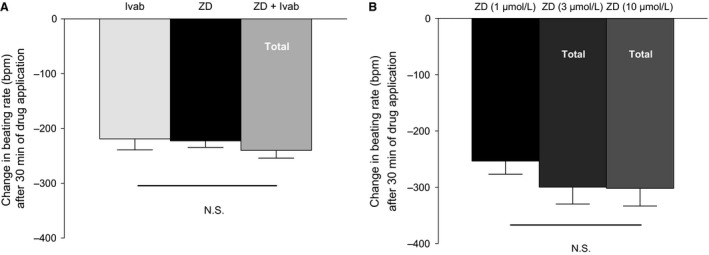
(A) The effect of I(f) inhibition by 1 *μ*mol/L ZD7288 (*n* = 9), 3 *μ*mol/L ivabradine (*n* = 10), and the combination of 1 *μ*mol/L ZD7288 + 3 *μ*mol/L ivabradine (*n* = 8, Total represents cumulative change from baseline), (B) 1 *μ*mol/L ZD7288 (*n* = 6), 3 *μ*mol/L ZD7288 (*n* = 6, Total represents cumulative change from baseline), and 10 *μ*mol/L ZD7288 (*n* = 6, Total represents cumulative change from baseline) on the spontaneous beating rate of mouse atrial preparations. Data are expressed as mean ± SEM,* n *= number of experiments. GraphPad prism (version 5.0) software was used to perform statistical analysis including Student's *t*‐test (significance level, *P *< 0.05).

Two questions might be raised concerning the use of ZD7288 to block I(f): how selective is ZD7288 at the concentration used (1 *μ*mol/L), and is blockade of I(f) complete at this concentration? The selectivity of ZD7288 I(f) inhibition is likely to be high at 1 *μ*mol/L since BoSmith et al. (1993) found this concentration to have little or no effect on Ca^2+^ and K^+^ currents. We explored whether the blockade of I(f) was complete by exposing the tissue to additional drugs to test for further reductions in beating rate, as might occur if there were significant residual I(f) in the presence of 1 *μ*mol/L ZD7288. Ivabradine is reported to be a selective I(f) blocker with little or no effect on other currents at 3 *μ*mol/L (Bois et al. 1996 & Bucchi et al. 2002). When ivabradine was added at 3 *μ*mol/L in the presence of 1 *μ*mol/L ZD7288 there was little or no further reduction in spontaneous beating rate (Fig. 3A, 30‐min preincubation with ZD7288 followed by a further 30‐min incubation with ivabradine). In this series of experiments, 1 *μ*mol/L ZD7288 decreased the spontaneous beating rate from 389 ± 18 bpm to 164 ± 14 bpm, a reduction of 225 ± 12 bpm, 57 ± 4%, *P *<* *0.05, *n *=* *9 (and similar to that reported for the first series of experiments above). Ivabradine alone (3 *μ*mol/L) showed a similar effect on spontaneous beating rate, causing a decrease from 378 ± 17 bpm to 159 ± 21 bpm, a reduction of 219 ± 20 bpm, 58 ± 5%, *P *<* *0.05, *n *=* *10. When 3 *μ*mol/L ivabradine was added in the presence of 1 *μ*mol/L ZD7288, there was little or no further change in beating rate (21 ± 5 bpm, *P *>* *0.05, *n *=* *8). Experiments were also carried out at higher ZD7288 concentrations (although it is recognized that increasing the concentration above 1 *μ*mol/L might reduce specificity as a consequence of additional effects on other channels; BoSmith et al. 1993). When the concentration of ZD7288 was sequentially increased from 1 to 3 to 10 *μ*mol/L, it was observed that there was little or no further decrease in beating rate (Fig. 3B, with each concentration being incubated for 30 min): in this series of experiments, 1 *μ*mol/L ZD7288 reduced the spontaneous beating rate by 253 ± 23 bpm (*P *<* *0.05, *n *=* *6), while increasing the concentration to 3 *μ*mol/L further reduced the beating rate by 46 ± 9 bpm (*P *<* *0.05, *n *=* *6) and subsequently increasing the concentration to 10 *μ*mol/L caused a small additional reduction of 2 ± 6 bpm (statistically insignificant, *n *=* *6). Taken together, these observations are consistent with the view that blockade of I(f) by ZD7288 is near complete at a concentration of 1* μ*mol/L (see Discussion for further consideration of this point).

**Figure 4:**
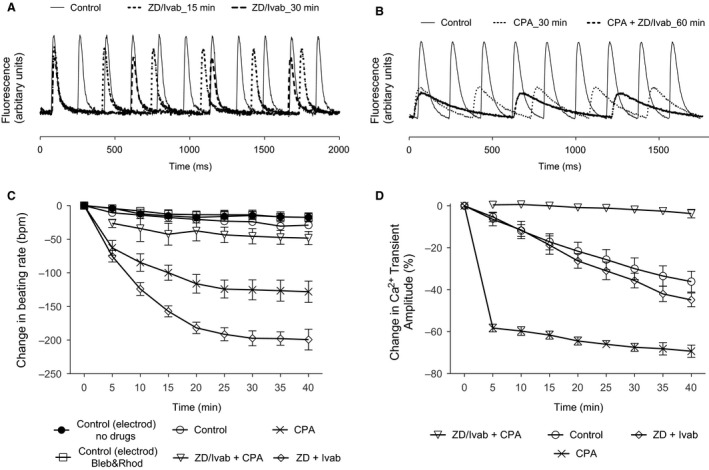
Representative traces of fluorescence Ca^2+^ transient measurements (Rhod‐2 as Ca^2+^ probe) from the SAN region are shown at different time periods. (A) Control and 3 *μ*mol/L ivabradine/1 *μ*mol/L ZD7288; (B) Control, 100 *μ*mol/L CPA, and 100 *μ*mol/L CPA in the presence of 3 *μ*mol/L ivabradine/1 *μ*mol/L ZD7288. The mean changes over time in rate of beating measured from the fluorescence Ca^2+^ transients recorded from the SAN region are shown in (C); the mean changes in amplitude of Ca^2+^ transients over the same time are shown in (*D*). There was a small reduction in rate under Control conditions over 40 min (open circles). There was a marked but slowly developing reduction in rate in the presence of 3 *μ*mol/L ivabradine/1 *μ*mol/L ZD7288 (open diamonds), and a slightly smaller progressive slowing of rate with 100 *μ*mol/L CPA (crosses; note that the effect of CPA on rate appeared to develop more slowly than the effect on amplitude, see text); the further reduction in rate when 3 *μ*mol/L ivabradine/1 *μ*mol/L ZD7288 was added after 100 *μ*mol/L CPA was small (open inverted triangles). Under Control conditions, and in the presence of 3 *μ*mol/L ivabradine/1 *μ*mol/L ZD7288, there was a progressive fall in the amplitude of Ca^2+^ transients that was thought primarily to reflect extrusion of Rhod‐2 (combined perhaps with photobleaching). The decline in the amplitude of Ca^2+^ transients was initially much faster with 100 *μ*mol/L CPA. The further decline in amplitude of Ca^2+^ transients was small when 3 *μ*mol/L ivabradine/1 *μ*mol/L ZD7288 was added in the presence of 100 *μ*mol/L CPA. Data are expressed as mean ± SEM. GraphPad prism (version 5.0) software was used to perform statistical analysis including Student's *t*‐test and two‐way analysis of variance (ANOVA), Bonferroni posttest (significance level, *P *< 0.05).

It was reported above that the reductions in rate caused by blockade of I(f) appeared to be less when SR function was suppressed. This apparent reduction in effect could occur if the activity of the Ca^2+^‐stimulated adenylyl cyclases AC1 and AC8 were to be at least in part elevated by Ca^2+^ released by the SR (Mattick et al. 2007) so that cAMP levels might be reduced following exposure to CPA. We therefore investigated the effects of blockade of I(f) under conditions in which cytosolic Ca^2+^ was measured using a fluorescent probe for Ca^2+^ (Rhod‐2, AM). Blebbistatin (10 *μ*mol/L) was added to suppress contraction and avoid contraction artifacts in the optical signal for Ca^2+^. In order to assess whether blebbistatin or Rhod‐2 had any effect on basal beating rate, an external electrode was used placed close to the SAN region to record the extracellular compound action potential signal. Forty‐minute incubation with blebbistatin (10 *μ*mol/L) together with Rhod‐2, AM (4 *μ*mol/L) was associated with a small fall in basal beating rate (17 ± 7 bpm, *n *=* *11), but this reduction in basal beating rate was not statistically different from that observed in the absence of Rhod‐2 and blebbistatin (16 ± 7 bpm, *n *=* *8, Fig. 4C) over the same time period. It therefore appears that blebbistatin and Rhod‐2 had little or no effect on spontaneous rate under these conditions.

Although probenecid can be used to reduce extrusion of fluorescent probe, this substance was avoided as it has been implicated to alter the physiology of Ca^2+^ handling (Koch et al. 2012). Instead of using drugs to reduce extrusion of Rhod‐2, the temperature was reduced to 34°C since even a small reduction in temperature is known to reduce extrusion of the Ca^2+^ probe. The basal beating rates were therefore slightly slower in experiments measuring Ca^2+^ transients than was reported above for the organ bath experiments at 37°C.

In experiments using Rhod‐2, the fall in beating rate measured over 40 min from intervals between Ca^2+^ transients was 29 ± 5 bpm or 10 ± 1%, *P < *0.05, *n *=* *6 (Fig. 4C*,* control). During this period the amplitude of the Ca^2+^ fluorescence transient fell by 36 ± 5%, *P *<* *0.05, *n *=* *6, presumably as a consequence of extrusion of the Ca^2+^ probe, and/or possible dye photobleaching (Fig. 4D, control).

The ivabradine/ZD7288 combination caused a reduction in beating rate of 199 ± 15 bpm or 62 ± 3%, *P *<* *0.05, *n *=* *9, after 40 min (Fig. 4C*,* Fig. 4A gives representative traces), similar to that observed in the absence of Rhod‐2 and blebbistatin (Fig. 1). Over the 40‐min period of exposure to the ivabradine/ZD7288 combination, the reduction in amplitude of the Ca^2+^ transient (at 40 min, 45 ± 3%) was not significantly different from the reduction observed in the absence of drugs (5–40 min, *P *>* *0.05, *n *=* *6, two‐way ANOVA, Bonferroni posttest, *n *=* *9 Fig. 4D). The similarity of the decline in amplitude of Ca^2+^ transients in the presence and absence of ivabradine/ZD7288 shows that there were no substantial effects of these drugs at the concentrations used on Ca^2+^ channels, or other aspects of Ca^2+^ handling. A small increase in the rate of decline of Ca^2+^ transients when ivabradine and ZD7288 were present cannot be ruled out, and might perhaps result from secondary consequences of the reduction in rate leading to changes in Ca^2+^ handling (see Discussion).

Although the decline of Ca^2+^ transient amplitude over time in the presence or absence ivabradine/ZD7288 was gradual, exposure to CPA caused a rapid reduction in Ca^2+^ transient amplitude over the first 5 min by 48 ± 1%, *P *<* *0.05, *n *=* *3, at 30 *μ*mol/L or by 57 ± 2%, *P *<* *0.05, *n *=* *6, at 100 *μ*mol/L (Fig. 4D, data for 30* μ*mol/L are not shown), presumably as a consequence of the suppression of SR function by inhibition of Ca^2+^ uptake by SERCA (Seidler et al. 1989). There was then a further gradual decline in amplitude after the first 5 min exposure to CPA, which is likely to have been at least in part caused by dye extrusion and/or photobleaching.

At least some of the effect of CPA on beating rate presumably results from a reduction in sodium–calcium exchange current associated with the reduced Ca^2+^ transient and suppression of local Ca^2+^ release events (Huser et al. 2000; Bogdanov et al. 2001). Such effects might be expected to develop rapidly over a similar time course to the initial reduction in Ca^2+^ transient amplitudes. There appeared to be a developing further reduction in rate over the time period from 5 to 30 min exposure to CPA (Fig. 4C): there was a significantly greater slowing at 30 than at 5 min (*P *<* *0.01, paired *t*‐test) and the data were well fitted by an exponential decay to a new steady rate of 129 ± 8 bpm with a time constant of 9.0 ± 1.8 min (although the effects on amplitude in Fig. 4D were substantially complete by 5 min). The slowing of beating rate in the presence of CPA could also in part reflect a reduction in activity of Ca^2+^‐stimulated adenylyl cyclases and consequent reduction in cAMP levels that regulate I(f). It seems possible that these additional effects might develop more slowly, perhaps because it takes time for the change in Ca^2+^ levels to be sensed and/or for the proposed reduction in activity of Ca^2+^‐stimulated adenylyl cyclases to lead to slow changes in cAMP levels that are at least in part the result of Ca^2+^‐dependent reduction of this enzyme activity.

In the presence of CPA (which alone caused a beating rate reduction of 129 ± 14 bpm from a resting beating rate of 299 ± 9 bpm to 170 ± 14 bpm, or 43 ± 5%, at the 30‐min period, *P *<* *0.05, *n *=* *6, Fig. 4A), the ivabradine/ZD7288 combination caused a further decline in beating rate (inverted triangles, Fig. 4C), but this decline was smaller (a further reduction of 48 ± 10 bpm or 19 ± 3%, *P <* 0.05, *n* = 6, at 70‐min period Fig. 4C than that caused by ivabradine/ZD7288 in the absence of CPA (199 ± 15 bpm or 62 ± 3%, *P < *0.05, *n* = 9, after 40 min). The observed reduced effect of ivabradine/ZD7288 under these conditions resembles the effects reported above (Fig. 1) for the organ bath experiments under similar conditions but without the Ca^2+^ probes.

### Effects of I(f) blockade and suppression of SR function on spontaneous beating rate in the presence of β‐adrenoceptor stimulation

We next investigated the changes in spontaneous beating rate induced by *β*‐adrenoceptor stimulation, under conditions in which the I(f) was blocked or SR function was suppressed. For these experiments, we returned to the simple organ bath preparation without Rhod‐2 or blebbistatin. In the absence of other compounds, isoprenaline caused a concentration‐dependent increase in beating rate with a maximum increase of 303 ± 10 bpm at 100 nmol/L. Several series of experiments were carried out with a variety of drug combinations. The initial aim was to block I(f) using 1 *μ*mol/L ZD7288 (30‐min preincubation during which the beating rate declined to a steady‐state plateau); the presence of ZD7288 reduced the isoprenaline‐induced increase in beating rate at each concentration, and the reductions were statistically significant (1 nmol/L, *P *<* *0.05, 3–30 nmol/L, *P *<* *0.01, 100 nmol/L, *P *<* *0.001, *n* = 6, two‐way ANOVA, Bonferroni posttest). The effects of a combination of 1 *μ*mol/L ZD7288 and 3 *μ*mol/L ivabradine (again allowing a 30‐min preincubation during which the beating rate declined to a steady‐state plateau) were similar those of 1 *μ*mol/L ZD7288 alone. However, it was clear that substantial effects of isoprenaline on beating rate remained even in the presence of I(f) blockade that was thought to be close to complete with the combination of I(f) blockers (see Discussion).

**Figure 5:**
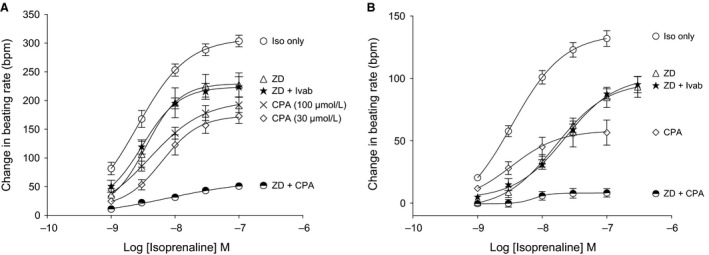
Modulation of the positive chronotropic action of the *β*‐adrenoceptor agonist, isoprenaline, in mouse spontaneously beating atrial preparation (A) by 100 *μ*mol/L CPA (*n* = 13), 1 *μ*mol/L ZD7288 (*n* = 6), 1 *μ*mol/L ZD7288 + 3* μ*mol/L ivabradine (*n* = 6), and 1 *μ*mol/L ZD7288 + 100 *μ*mol/L CPA (*n* = 10). Similar observations in guinea pig spontaneously beating atrial preparations are shown in (B) for Control (*n* = 6), 30 *μ*mol/L CPA (*n* = 6), 1 *μ*mol/L ZD7288 (*n* = 6), 1 *μ*mol/L ZD7288 + 3 *μ*mol/L ivabradine (*n* = 6), and 1 *μ*mol/L ZD7288 + 30 *μ*mol/L CPA (*n* = 6). Data are expressed as mean ± SEM,* n *= number of experiments. GraphPad prism (version 5.0) software was used to perform statistical analysis including two‐way ANOVA, Bonferroni posttest (significance level, *P *< 0.05).

CPA (30 and 100 *μ*mol/L, again allowing a 30‐min preincubation during which the beating rate declined to a steady‐state plateau) also depressed the log (concentration) – response to isoprenaline (30 and 100 *μ*mol/L CPA: 1 nmol/L, *P* < 0.05, 3–100 nmol/L, *P *<* *0.001, *n *=* *13, two‐way ANOVA, Bonferroni posttest). The effects of these drugs that suppress SR function to cause a depression of the slope and maximum of the log(concentration)–response to isoprenaline were slightly greater than the effects of blockade of I(f) alone (Fig. 5B).

When the ZD7288 to block I(f) and CPA to depress SR function were used together (30‐min preincubation of a tissue with CPA, followed by additional 30 min incubation with ZD7288 in order to reach a further reduced plateau steady beating rate: 1–100 nmol/L, *P *<* *0.001, *n *=* *10, two‐way ANOVA, Bonferroni posttest), there was a substantial further depression of the log(concentration)–response curve to isoprenaline relative to either CPA or ZD7288 when used alone. The change in beating rate at the maximum concentration of 100 nmol/L was substantially reduced to 51 ± 2 bpm, *n *=* *10 (Fig. 5A) under these conditions.

When similar experiments were performed in guinea pig spontaneously beating atrial preparations the effects of the drugs used in the experiments above were broadly similar to those seen in mouse preparations. ZD7288 (1 *μ*mol/L) alone (preincubating for 30 min) again depressed the log(concentration)–response curve to isoprenaline, so that the change in beating rate was less at all concentrations studied (3–30 nmol/L, *P *<* *0.001, *n *=* *6, two‐way ANOVA, Bonferroni posttest). The effects of a combination of 1 *μ*mol/L ZD7288 and 3 *μ*mol/L ivabradine (preincubating for 30 min to reach a steady‐state reduction) were similar those of 1 *μ*mol/L ZD7288 alone (3–30 nmol/L, *P *<* *0.001, *n *=* *6, two‐way ANOVA, Bonferroni posttest). However, substantial increases in spontaneous beating rate in response to isoprenaline remained in the presence of these drugs to block I(f). At the highest concentration tested, 100 nmol/L, isoprenaline increased the beating rate by 85 ± 16 bpm in the presence of 1 *μ*mol/L ZD7288, and by 88 ± 15 bpm in the presence of 1 *μ*mol/L ZD7288 followed by 3 *μ*mol/L ivabradine, compared with the control increase of 132 ± 21 bpm. The difference between the curves produced in the presence of 1 *μ*mol/L ZD7288 compared to 1 *μ*mol/L ZD7288 followed by 3 *μ*mol/L ivabradine was not statistically significant. CPA (30 *μ*mol/L, preincubated for 30 min) caused a greater depression of the maximum effect of isoprenaline at 100 nmol/L by 57 ± 9 bpm (3 nmol/L, *P *<* *0.01, *n *=* *13, 10–100 nmol/L, *P *<* *0.001, *n *=* *13, two‐way ANOVA, Bonferroni posttest) than was observed with ZD7288 with or without ivabradine. Finally, the maximum effect of isoprenaline at 100 nmol/L in the presence of 1 *μ*mol/L ZD7288 and 30 *μ*mol/L CPA (with each drug being incubated for 30 min) was substantially suppressed to 8 ± 2 bpm compared to the control of 132 ± 21 bmp (Fig. 5B). The broad pattern of the effects of the drug combinations in guinea pig (compare Fig 5A and B) was therefore very similar to that of the same drugs in mice even though the maximal bpm changes differ for the various drug combinations.

## Discussion

In discussing the implications and limitations of the findings presented here, consideration should be given to the selectivity of drug treatments used. It is also important to note that it could be argued that in a complex system the conclusions that can be drawn from selective alteration of one variable are limited (see, e.g., Yaniv et al. 2013, 2014). Nonetheless, the observations reported here do impose constraints on models of the initiation of spontaneous activity by the SA node, and the combined results can be taken to imply that both SR function and I(f) currents make substantial contributions to pacemaker activity. This was found to be the case both at rest (in the absence of autonomic neurotransmitters and hormones) and in the presence of the *β*‐adrenoceptor agonist isoprenaline.

The recent experiments of Yaniv et al. (2013) investigate the effects of 3 *μ*mol/L ivabradine in rabbit‐isolated SA node myocytes on Ca^2+^ cycling as well as I(f), and conclude that there are effects on Ca^2+^ cycling under the conditions of their experiments that contribute to the slowing of spontaneous rate. In particular, ivabradine was found to reduce SR Ca^2+^ load. This was tested with a rapid caffeine “spritz” and 3 *μ*mol/L ivabradine was reported to reduce the amplitude of the caffeine‐induced Ca^2+^ transient by 13% (but see also Yaniv et al. 2014; in which the effects of ivabradine on SR Ca^2+^ content were reported only to become significant at concentrations of 10 *μ*mol/L and above). An indirect effect of a bradycardic agent on SR Ca^2+^ is not surprising as discussed above (since even when spontaneous rate is slowed by a completely selective I(f) blocker the fall in heart rate will reduce the number of action potentials per unit time that permit Ca^2+^ influx via voltage‐gated Ca^2+^ channels, and this reduction in Ca^2+^ influx would be expected to cause diminished Ca^2+^ uptake by the SR). Although an effect of this kind is expected, it appears that under the conditions of our experiments any effect was small (and not statistically significant) for our observations using the 3 *μ*mol/L ivabradine and 1 *μ*mol/L ZD7288 combination in mouse intact SA preparations, since there was little or no difference in the decline of the amplitude of Ca^2+^ transients in the absence and presence of these drugs over a 30‐min exposure period, even though these drugs caused an approximate halving of spontaneous rate. Future experiments will be required to investigate whether species differences (e.g., in basal heart rates and/or the balance of different ionic currents) might account for the apparent discrepancy between our observations and those of Yaniv et al. (2013).

An important observation presented here is that the substantial effects of isoprenaline remain in the presence of I(f) blockers, ZD7822 or ivabradine. This is unlikely to result from incomplete blockade of I(f) by these drugs at the concentrations used as discussed later. The effects of ZD7822 in combination with CPA profoundly suppress changes in spontaneous beating rate caused by *β*‐adrenoceptor stimulation, and these observations are most easily explained if there are important contributions of both I(f) and SR‐dependent mechanisms to the *β*‐adrenoceptor pathways (although overlap of these mechanisms is not excluded as discussed further below). Work with the canine SA node using fluorescent probes for Ca^2+^ also led to the conclusion that both I(f) and SR‐dependent mechanisms contribute to pacemaker activity (Joung et al. 2009; Gao et al. 2010), although these experiments did not explore the catecholamine effects over a broad concentration range.

Using electrophysiological approaches to test for the effectiveness of I(f) blockade is technically difficult particularly with agents such as ivabradine that are thought to be “use dependent” and which take a long time to reach a steady‐state effect (observed to be 30 min for atrial preparations, and therefore blockade may be underestimated if the exposure time is too short). “Rundown” of currents can also be a problem over long time periods. The first paper on ZD7288 quotes 74 ± 4% blockade at 1 *μ*mol/L (BoSmith et al. 1993), but this may be an underestimate because of the difficulty of making electrophysiological measurements over a sufficiently long time to achieve steady state. Under the conditions of our experiments, 3 *μ*mol/L ivabradine caused a similar (58 ± 5%) reduction in beating rate and the degree of block caused by this drug is expected to be at least as high as that for ZD7288 (Bois et al. 1996; Bucchi et al. 2002).

As a starting point, we assume first that ZD7288 and ivabradine occupy the same site on the I(f) channel, and that each drug separately occupies 75% of these sites under the conditions of our experiments (consistent with the 74 ± 4% blockade reported by BoSmith et al. 1993; and taking the similar effects of ivabradine under the conditions of our experiments to indicate that this drug also occupies a similar fraction of sites/channels at the concentration used). If the fraction of sites blocked = [D]/([D] + K_D_), where [D] is the drug concentration and K_D_ is the dissociation equilibrium constant for a bimolecular binding reaction of the drug to the site/channel, then 75% block would be expected to occur when the concentration of each drug is three times greater than K_D_ under the conditions of our experiments (while also bearing in mind that, as argued above, the fractions of sites blocked that have been used for this calculation might well be underestimates). Following the analysis of Jarvis and Thompson (2013) who compared ion channel blockade by drugs occupying the same or different sites, and assuming first that each drug shows mutually exclusive binding to the same site on the I(f) channel, the fraction of channels occupied by the two drugs combined would be (3 + 3)/(3 + 3 + 1) or 86%. The authors also calculated the consequence of the second case where the two blocking drugs occupy different sites. In this case, if 1 *μ*mol/L ZD7288 blocks 75% of the channels leaving a residual conductance of 0.25 of the total, and if 3 *μ*mol/L ivabradine binds at a different site, and also cause 75% block (0.25 residual conductance), the two drugs together would be predicted to leave a residual conductance of unblocked channels of 0.25 × 0.25 = 0.0625 of the total conductance or close to 94% blockade. If either of these estimates for blockade were underestimates (as may be the case as a consequence of the difficulty in carrying out prolonged electrophysiological studies of ion channel blockade), the estimates for combined block are even greater (for both the case of identical and different binding sites). In other words, the combination of 1 *μ*mol/L ZD7288 and 3 *μ*mol/L ivabradine may approach near complete blockade of I(f). The observation that there was little or no difference between the effects observed following 1 *μ*mol/L ZD7288, 3 *μ*mol/L ivabradine, or a combination of these two drugs may be rationalized by the proposal that 1 *μ*mol/L ZD7288 and 3 *μ*mol/L ivabradine each cause almost complete blockade of I(f) under these conditions, and that there is little further effect when the two drugs are combined. This view is also supported by the modest changes in spontaneous rate observed under these conditions when the concentration of ZD7288 was increased from 1 to 3 and then 10 *μ*mol/L. The rate changes that we see in mouse when we believe little or no I(f) remains unblocked are remarkably similar to those observed by Mesirca et al. (2014) using genetic techniques to silence I(f) channels.

If these arguments are correct, their implications for pacemaker mechanisms underlying resting beating rate (in the absence of autonomic transmitters or hormones) can be considered. If very little I(f) remains in the presence of ivabradine and ZD7288, the observation that in mice the beating rate is reduced by approximately 50% by these drugs may indicate that residual mechanisms other than I(f) play a major role under these conditions. Such additional mechanisms could include both “membrane clock” mechanisms other than I(f), and “calcium clock” mechanisms. The membrane clock mechanisms include the importance of deactivating voltage‐gated K channels (together with an absence of a “stabilizing” resting K channel activity), sustained inward current, voltage‐gated Ca^2+^ channels and NCX (see Capel and Terrar 2015a,b). Voltage‐gated Ca^2+^ channels and NCX are also important for “calcium clock” mechanisms. The role of the SR is in part to determine the timing of Ca^2+^ release and uptake mechanisms, but there may be additional longer term effects of Ca^2+^ released from the SR (see below).

The observation that the effects of I(f) blockers on resting beating rates were less (measured as both % and bpm changes) in the presence than in the absence of ryanodine or CPA is consistent with a reduced contribution of I(f) under these conditions. Such reduced contribution of I(f) when SR Ca^2+^ release is suppressed could arise if SR‐derived Ca^2+^ were to activate Ca^2+^‐stimulated adenylyl cyclases such as AC1 or AC8, since this would lead to reduced levels of cAMP modulating I(f). Evidence supporting the contribution of such a pathway is provided by Mattick et al. (2007) and Younes et al. (2008). Functional interaction between AC1 and HCN2 has also been shown in myocytes in which HCN2 was coexpressed with AC1, AC6, or GFP. When Ca^2+^‐stimulated AC1 was coexpressed with HCN2, the I(f) current was activated at potentials approximately 10 mV more depolarized than when HCN2 expression was coupled with AC6 or GFP (Kryukova et al. 2012).

CPA is used at 100 *μ*mol/L and 30 *μ*mol/L, and it seems possible that nonspecific effects might occur, particularly at the higher concentration, but the observations with 30 *μ*mol/L were remarkably similar to those at 100 *μ*mol/L, consistent with a maximal drug actions. It is our experience that concentrations of blocking agents required for intact multicellular atrial preparations frequently appear to be higher than those required in isolated myocytes, perhaps because of barriers to permeation or other impediments to tissue penetration (see Rigg and Terrar 1996; Rigg et al. 2000).

The above arguments might be extended to consideration of the effects of stimulation of *β*‐adrenoceptors by isoprenaline. In terms of the contribution of I(f) to the positive chronotropic effect, isoprenaline will have effects via a direct G‐protein‐mediated stimulation of adenylyl cyclases, and possible additional effects that could arise as a consequence of an increased amplitude of the Ca^2+^ transient (Rigg et al. 2000) that might in turn stimulate the adenylyl cyclases AC1 and/or AC8. Such a mechanism is also supported by the experiments of Kryukova et al. 2012 mentioned above: in myocytes coexpressing HCN2 and AC1, isoprenaline caused a further positive shift of the activation curve for I(f) and this effect was prevented by the Ca^2+^ chelator BAPTA.

Some of the effects of CPA to suppress the log(concentration)–response curve to isoprenaline and reduce the maximum chronotropic response might be attributable to a reduced I(f) as a consequence of the reduced amplitude of the Ca^2+^ transient and consequent reduced contribution of Ca^2+^‐stimulated AC1 and/or AC8. This hypothesis receives important support from the observations in which Ca^2+^ transients were measured using Rhod‐2. It is clear that the majority of the effect of CPA on the amplitude of Ca^2+^ transients developed within 5 min, and any effects on spontaneous rate that depend directly on changes in Ca^2+^ transients or local Ca^2+^ release events (e.g., involving direct influences of NCX currents) would be expected to be apparent on this time scale. In contrast to the rapid effect of CPA on the amplitude of Ca^2+^ transients, it was observed that the full effect of CPA on spontaneous rate (determined from the intervals between Ca^2+^ transients) took much longer to develop over a period of approximately 30 min. These observations are consistent with a slow development of effects of changed Ca^2+^ transients on Ca^2+^‐stimulated enzymes including AC1 and AC8. The slow development of effect could arise from a slow readjustment of cAMP levels as a consequence of changes in enzyme activity. The magnitude of I(f), and consequently the influence of this current on spontaneous rate, would be expected to closely follow the reduced levels of cAMP, but additional effects as a consequence of altered PKA function influencing other ion channels would also be predicted. It therefore seems likely that suppression of SR function will have effects on a number of pathways involved in the positive chronotropic response that are additional to any effects of I(f), as discussed in more detail below.

When I(f) was blocked by ZD7288 and ivabradine in various combinations, it appeared that substantial increases in beating rate in response to *β*‐adrenoceptor stimulation remained. From the arguments above concerning the effectiveness of these drugs it seems unlikely that the continuing effects of *β*‐adrenoceptor stimulation can be explained by effects on a large component of I(f) that remains unblocked. An additional experimental observation that supports the contention that the remaining effect of isoprenaline in the presence of the ZD7288 and ivabradine combination is not primarily a consequence of effects of isoprenaline on residual I(f) is the observation that so little of the effect of isoprenaline remained when the ZD7288 and ivabradine combination was supplemented with CPA.

The substantial additional effects of CPA in addition to blockade of I(f) are best explained by effects of CPA that depend on SR Ca^2+^, but do not involve this current. Targets for *β*‐adrenoceptor stimulatory mechanisms that could involve Ca^2+^‐stimulated adenylyl cyclases but not I(f) include pathways that are influenced by PKA following changes in cAMP levels. These include voltage‐gated Ca^2+^ channels, NCX (at least indirectly as a consequence of increased subsarcolemmal Ca^2+^), phospholamban/SERCA, voltage‐gated K channels, and sustained inward current channels (Capel and Terrar 2015b). There may also be effects via CaMKII that involve SR‐released Ca^2+^ (Wu et al. 2014). In addition, SR‐dependent effects on L‐type Ca^2+^ currents thought to act via AC1 and/or AC8 in atrial myocytes have been recently described (Collins and Terrar 2012).

In summary, our results are consistent with contributions from both I(f) and SR‐dependent mechanisms to pacemaker activity at rest and during *β*‐adrenoceptor stimulation. Some of the effects of SR‐released Ca^2+^ can influence I(f) through activation of Ca^2+^‐stimulated AC1 and/or AC8, but there appear to be substantial additional SR‐dependent effects that are unrelated to I(f).

## Conflict of Interest

None declared.

## References

[phy212561-bib-0001] Bogdanov, K. Y. , T. M. Vinogradova , and E. G. Lakatta . 2001 Sinoatrial nodal cell ryanodine receptor and Na^+^‐Ca^2+^ exchanger: molecular partners in pacemaker regulation. Circ. Res. 88:1254–1258. 11420301 10.1161/hh1201.092095

[phy212561-bib-0002] Bois, P. , J. Bescond , B. Renaudon , and J. Lenfant . 1996 Mode of action of bradycardic agent, S 16257, on ionic currents of rabbit sinoatrial node cells. Br. J. Pharmacol. 118:1051–1057. 8799581 10.1111/j.1476-5381.1996.tb15505.x PMC1909508

[phy212561-bib-0003] BoSmith, R. E. , I. Briggs , and N. C. Sturgess . 1993 Inhibitory actions of ZENECA ZD7288 on whole‐cell hyperpolarization activated inward current I(f) in guinea‐pig dissociated sinoatrial node cells. Br. J. Pharmacol. 110:343–349. 7693281 10.1111/j.1476-5381.1993.tb13815.x PMC2176028

[phy212561-bib-0004] Bucchi, A. , M. Baruscotti , and D. DiFrancesco . 2002 Current‐dependent block of rabbit sino‐atrial node I(f) channels by ivabradine. J. Gen. Physiol. 120:1–13. 12084770 10.1085/jgp.20028593 PMC2238187

[phy212561-bib-0005] Capel, R. A. , and D. A. Terrar . 2015a Cytosolic calcium ions exert a major influence on the firing rate and maintenance of pacemaker activity in guinea‐pig sinus node. Front. Physiol. 23:1–8. 10.3389/fphys.2015.00023 PMC4322845 25713538

[phy212561-bib-0006] Capel, R. A. , and D. A. Terrar . 2015b The importance of Ca^2+^‐dependent mechanisms for the initiation of the heartbeat. Front. Physiol. 80:1–34. 10.3389/fphys.2015.00080 PMC4373508 25859219

[phy212561-bib-0007] Collins, T. P. , and D. A. Terrar . 2012 Ca2 + ‐ stimulated adenylyl cyclases regulate the L‐type Ca2 + current in guinea‐pig atrial myocytes. J. Physiol. (Lond.) 590:1881–1893. 22351635 10.1113/jphysiol.2011.227066 PMC3573310

[phy212561-bib-0008] DiFrancesco, D. 2010 The role of the funny current in pacemaker activity. Circ. Res. 106:434–446. 20167941 10.1161/CIRCRESAHA.109.208041

[phy212561-bib-0010] Gao, Z. , B. Chen , A. J. Mei‐ling , Y. Wu , X. Guan , and O. M. Koval . 2010 I_f_ and SR Ca^2+^ release both contribute to pacemaker activity in canine sinoatrial node cells. J. Mol. Cell. Cardiol. 49:33–40. 20380837 10.1016/j.yjmcc.2010.03.019 PMC2883640

[phy212561-bib-0011] Grupp, G. , I. Grupp , C. Johnson , M. Matlib , W. Rouslin , A. Schwartz , et al. 1980 Effects of RMI 12330A, a new inhibitor of adenylate cyclase on myocardial function and subcellular activity. Br. J. Pharmacol. 70:429–442. 6254599 10.1111/j.1476-5381.1980.tb08721.x PMC2044352

[phy212561-bib-0012] Himeno, Y. , F. Toyoda , H. Satoh , A. Amano , C. Y. Cha , H. Matsuura , et al. 2011 Minor contribution of cytosolic Ca2 transients to the pacemaker rhythm in guinea pig sinoatrial node cells. Am. J. Physiol. Heart Circ. Physiol. 300:H251–H261. 20952667 10.1152/ajpheart.00764.2010

[phy212561-bib-0013] Huser, J. , L. A. Blatter , and S. L. Lipsius . 2000 Intracellular Ca^2+^ release contributes to automaticity in cat atrial pacemaker cells. J. Physiol. (Lond.) 524:415–422. 10766922 10.1111/j.1469-7793.2000.00415.x PMC2269880

[phy212561-bib-0014] Jarvis, G. E. , and A. J. Thompson . 2013 A golden approach to ion channel inhibition. Trends Pharmacol. Sci. 34:481–488. 23972927 10.1016/j.tips.2013.07.004 PMC3769878

[phy212561-bib-0015] Joung, B. , L. Tang , M. Maruyama , S. Han , Z. Chen , M. Stucky , et al. 2009 Intracellular calcium dynamics and acceleration of sinus rhythm by *β*‐adrenergic stimulation. Circulation 119:788–796. 19188501 10.1161/CIRCULATIONAHA.108.817379 PMC2735196

[phy212561-bib-0016] Kanda, N. , and S. Watanabe . 2001 Regulatory roles of adenylate cyclase and cyclic nucleotide phosphodiesterases 1 and 4 in interleukin‐13 production by activated human T cells. Biochem. Pharmacol. 62:495–507. 11448460 10.1016/s0006-2952(01)00688-8

[phy212561-bib-0017] Koch, S. E. , X. Gao , L. Haar , M. Jiang , V. M. Lasko , N. Robbins , et al. 2012 Probenecid: Novel use as a non‐Injurious Positive Inotrope Acting via Cardiac TRPV2 Stimulation. J. Mol. Cell. Cardiol. 53:134–144. 22561103 10.1016/j.yjmcc.2012.04.011 PMC3372642

[phy212561-bib-0018] Kryukova, Y. N. , L. Protas , and R. B. Robinson . 2012 Ca^2+^ ‐activated adenylyl cyclase 1 introduces Ca 2 ‐dependence to beta‐adrenergic stimulation of HCN2 current. J. Mol. Cell. Cardiol. 52:1233–1239. 22484253 10.1016/j.yjmcc.2012.03.010 PMC3348422

[phy212561-bib-0019] Lakatta, E. G. , and D. DiFrancesco . 2009 What keeps us ticking: a funny current, a calcium clock, or both? J. Mol. Cell. Cardiol. 47:157–170. 19361514 10.1016/j.yjmcc.2009.03.022 PMC4554526

[phy212561-bib-0020] Lakatta, E. G. , V. A. Maltsev , and T. M. Vinogradova . 2010 A coupled SYSTEM of intracellular Ca2 clocks and surface membrane voltage clocks controls the timekeeping mechanism of the heart's pacemaker. Circ. Res. 106:659–673. 20203315 10.1161/CIRCRESAHA.109.206078 PMC2837285

[phy212561-bib-0021] Mattick, P. , J. Parrington , E. Odia , A. Simpson , T. Collins , and D. Terrar . 2007 Ca2 ‐stimulated adenylyl cyclase isoform AC1 is preferentially expressed in guinea‐pig sino‐atrial node cells and modulates the If pacemaker current. J. Physiol. (Lond.) 582:1195–1203. 17540702 10.1113/jphysiol.2007.133439 PMC2075242

[phy212561-bib-0022] Mesirca, P. , J. Alig , A. G. Torrente , J. C. Müller , L. Marger , A. Rollin , et al. 2014 Cardiac arrhythmia induced by genetic silencing of ‘funny’(f) channels is rescued by GIRK4 inactivation. Nat. Commun. 5:1–15. 10.1038/ncomms5664 PMC4207211 25144323

[phy212561-bib-0023] Rigg, L. , and D. A. Terrar . 1996 Possible role of calcium release from the sarcoplasmic reticulum in pacemaking in guinea‐pig sino‐atrial node. Exp. Physiol. 81:877–880. 8889484 10.1113/expphysiol.1996.sp003983

[phy212561-bib-0024] Rigg, L. , B. M. Heath , Y. Cui , and D. A. Terrar . 2000 Localisation and functional significance of ryanodine receptors during *β*‐adrenoceptor stimulation in the guinea‐pig sino‐atrial node. Cardiovasc. Res. 48:254–264. 11054472 10.1016/s0008-6363(00)00153-x

[phy212561-bib-0025] Seidler, N. W. , I. Jona , M. Vegh , and A. Martonosi . 1989 Cyclopiazonic acid is a specific inhibitor of the Ca^2+^‐ATPase of sarcoplasmic reticulum. J. Biol. Chem. 264:17816–17823. 2530215

[phy212561-bib-0026] Vinogradova, T. M. , K. Y. Bogdanov , and E. G. Lakatta . 2002 *β*‐Adrenergic stimulation modulates ryanodine receptor Ca2 release during diastolic depolarization to accelerate pacemaker activity in rabbit sinoatrial nodal cells. Circ. Res. 90:73–79. 11786521 10.1161/hh0102.102271

[phy212561-bib-0027] Vinogradova, T. M. , Y. Y. Zhou , V. Maltsev , A. Lyashkov , M. Stern , and E. G. Lakatta . 2004 Rhythmic ryanodine receptor Ca2 + releases during diastolic depolarization of sinoatrial pacemaker cells do not require membrane depolarization. Circ. Res. 94:802–809. 14963011 10.1161/01.RES.0000122045.55331.0F

[phy212561-bib-0029] Wu, Y. , E. Mark , and M. E. Anderson . 2014 CaMKII in sinoatrial node physiology and dysfunction. Front. Pharmacol. 5:1–6. 24672485 10.3389/fphar.2014.00048 PMC3957193

[phy212561-bib-0030] Yaniv, Y. , S. Sirenko , B. D. Ziman , H. A. Spurgeon , V. A. Maltsev , and E. G. Lakatta . 2013 New evidence for coupled clock regulation of the normal automaticity of sinoatrial nodal pacemaker cells: bradycardic effects of ivabradine are linked to suppression of intracellular Ca^2+^ cycling. J. Mol. Cell. Cardiol. 62:80–89. 23651631 10.1016/j.yjmcc.2013.04.026 PMC3735682

[phy212561-bib-0031] Yaniv, Y. , A. E. Lyashkov , S. Sirenko , Y. Okamoto , T. R. Guiriba , B. D. Ziman , et al. 2014 Stochasticity intrinsic to coupled‐clock mechanisms underlies beat‐to‐beat variability of spontaneous action potential firing in sinoatrial node pacemaker cells. J. Mol. Cell. Cardiol. 77:1–10. 25257916 10.1016/j.yjmcc.2014.09.008 PMC4312254

[phy212561-bib-0032] Yaniv, Y. , E. G. Lakatta , and V. A. Maltsev . 2015 From two competing oscillators to one coupled‐clock pacemaker cell system. Front. Physiol. 6:1–8. 25741284 10.3389/fphys.2015.00028 PMC4327306

[phy212561-bib-0200] Younes, A. , A. E. Lyashkov , D. Graham , A. Sheydina , M. V. Volkova , M. Mitsak , et al. 2008 Ca(2+) ‐stimulated basal adenylyl cyclase activity localization in membrane lipid microdomains of cardiac sinoatrial nodal pacemaker cells. J. Biol. Chem. 283:14461–14468. 18356168 10.1074/jbc.M707540200 PMC2386925

